# Socioeconomic Determinants of Universal Health Coverage in the Asian Region

**DOI:** 10.3390/ijerph19042376

**Published:** 2022-02-18

**Authors:** Tomoyuki Takura, Hiroko Miura

**Affiliations:** 1 Department of Healthcare Economics and Health Policy, Graduate School of Medicine, The University of Tokyo, Tokyo 113-8654, Japan; 2 Division of Disease Control and Epidemiology, School of Dentistry, Health Sciences University of Hokkaido, Ishikari 061-0293, Japan; hmiura@hoku-iryo-u.ac.jp

**Keywords:** universal health coverage, service coverage index, gross domestic product, health expenditure, poverty, population, panel data analysis, performance

## Abstract

The World Health Organization (WHO) states that examining medical financial systems is the most important process in evaluating universal health coverage (UHC). This study used the service coverage index (SCI) as a proxy of the progress toward UHC in eleven Asian countries. We employed a fixed-effects regression model to analyze panel data from 2015 to 2017, to explain the interrelationship between the SCI and major socioeconomic indicators. We also conducted a performance analysis (ratio of achieved SCI level to gross domestic product (GDP) or health expenditure displacement) to examine the balance between the degree of achievements related to UHC and a country’s economic level. The results showed that GDP and health expenditure were significantly positively correlated with the SCI (*p* < 0.01). The panel data analysis results showed that GDP per capita was a factor that greatly influenced the SCI as well as poverty (partial regression coefficient: 0.0017, 95% CI: 0.0013–0.0021). The results of the performance analysis showed that the Philippines had the highest scores (GDP: 1.84 SCI score/USD per capita, health expenditure: 1.04 SCI score/USD per capita) and South Korea the lowest. We conclude that socioeconomic factors, such as GDP, health expenditure, unemployment, poverty, and population influence the progress of UHC, regardless of system maturity or geographic characteristics.

## 1. Introduction

The Sustainable Development Goals (SDGs), ratified by all United Nations member States in 2015, consist of 17 goals and 169 specific targets to be achieved by 2030—the aim of which is to realize a world free of poverty, hunger, and disease [1,2,3,4]. Of these goals, SDG 3 consists of thirteen targets related to the theme of “health and welfare for all.” The other 16 goals are also related to health or are positioned to contribute indirectly to health. The SDGs aim to “leave no one behind” and are international targets applicable to both developing and advanced countries. Universal health coverage (UHC) is a concept that includes (1) the protection from financial risks for all, (2) access to quality primary health services, and (3) access to essential medicines and vaccines that are safe, effective, high quality, and inexpensive. Target 3.8 of SDG 3, which involves achieving UHC and health improvement worldwide, is positioned as the most crucial task of the World Health Organization (WHO) [5].

UHC index measurement’s approaches and definitions evolved between 2015 and 2019, and the index is now used in every global monitoring report [6]. Comparison of UHC progress between regions and countries is now possible. Additionally, the service coverage index (SCI) has been calculated as a single number (score) since the late 2010s, improving comparability between nations. While it is now possible to compare the performance of different countries, global monitoring alone is insufficient to guide policymaking [7]. Therefore, each country is encouraged to develop a country-specific framework based on the global one. To achieve this, it is necessary to analyze the relationship between the environmental factors surrounding medical care and progress toward UHC. From this, it is possible to develop a predictive model for policy selection.

Problems regarding medical financial systems constitute a significant challenge to achieving UHC. The WHO emphasizes that a developed healthcare financial system that removes financial constraints on access to health services is essential [8,9]. Several past studies suggest that UHC is more likely to be achieved if patients’ out-of-pocket medical costs are low [10]. In a recent study, 46% of the 49 sampled countries showed a lack of financial resources as a major constraint on UHC deployment [11]. In addition, a lack of functional services and the inability of political leadership to support a national health system was shown to be the second constraint in 40% of countries.

It has been pointed out that even in the Asian region, the biggest constraint on the spread of UHC is healthcare capacity/affordability [12]. Other constraints include cultural factors, the lack of political will to implement UHC, and the lack of evidence-based evaluation (relying only on experts’ opinions). For example, a joint research program was undertaken by Japan and the World Bank wherein a qualitative study was conducted to evaluate health policy and health programs in eleven countries, including Japan. The results showed that strong political leadership is essential for achieving UHC [13]. Thus, extensive evidence is needed to promote strong UHC policy leadership and facilitate consensus building among stakeholders.

Previous related reports on policy trends reveal that rational decision-making that considers the economics of medicine is essential in shaping future medical policy [14]. Moreover, when aiming for sustainable and long-term system management, the value of this perspective is expected to increase. Based on the above, a quantitative analysis of the effects of health economic factors on UHC is required for discussions on the spread and development of UHC in the Asian region. However, there has been insufficient research on the relationship between UHC levels and economic factors, not only in the Asian region but also globally.

Therefore, in this study, we explored the relationship between SCI and major socioeconomic indicators to establish the relationship between UHC levels and economic factors, and we investigated the significance of this relationship through performance analysis. The aim of this study was threefold. We sought, first, to clarify the impact of socioeconomic factors on UHC development; second, to perform socioeconomic factor analysis considering the constituent factors of UHC; and third, to identify performance evaluation methods that take into account the socioeconomic characteristics of each country. These findings are expected to aid each country in navigating the future development of UHC.

## 2. Methods

### 2.1. Survey Design

UHC is a broad concept spanning fields such as politics, economics, medical care, and ethics, and covering complex systems involving multiple factors. Because of these varied characteristics, an interdisciplinary empirical research approach is needed. However, there is a limit to the immediate applicability of conventional research design (question setting and analysis method) when attempting this type of research. Therefore, to advance this research field, we base our approach on the concept of system design, which first focuses on important elements and then accumulates systematic arrangements. In sublimating UHC research to a comprehensive level, an approach that focuses on economic factors (which are often discussed only at a cursory level) and provides feedback while considering multiple research reports is needed. Based on the above, we conducted a preliminary study that analyzed the impact of socioeconomic factors on the SCI, which is used as a proxy for estimating the progress of UHC.

Before beginning this study, we reviewed previous analyses of socioeconomic factors that influence UHC progress using a general-purpose database (PubMed), searching for studies published since 2000. From this survey, we identified five reports on related themes. Among these, four reports discussed the actual situation regarding out-of-pocket of medical expenses and poverty from a social-system perspective [15,16,17,18]. One of the reports discussed the relationship between the UHC index and health spending [18]. In addition, there was one review article related to the cost-effectiveness analysis (CEA) of health care systems [19]. Two reports targeting the Asian region were cited in the review (Myanmar and Bangladesh). The main limitation reported in these studies was the lack of panel data analyses or CEA-based empirical studies, and a socioeconomic analysis of factors influencing the progress of UHC was suggested as an important subject for future research.

The Asian region targeted by this study is diverse, and the UHC-related and socioeconomic data for the region have the characteristics of complex systems. Thus, to discuss the displacement ratio with a small sample size, simple regression analysis and a performance analysis—an integrated indicator in which the indexes interfere with each other—was conducted using logarithmic transformation to reduce variance and outliers. Conversely, because we set up a hypothetical model in which the elastic force is constant, we found it difficult to reflect the meaning of each baseline in terms of factors such as institutional maturity and population composition. Therefore, we discussed the effect of the aging rate on performance by showing the results of calculations with non-logarithmic transformation. Furthermore, in multivariate analysis, we set a model that considers the unobservable characteristics peculiar to each country.

With the above approaches, two outcomes with different durations and methods are obtained. These outcomes may provide policymakers with information that allows for the discussion from various perspectives. In the future, policymakers are expected to take advantage of these multifaceted causal inference indicators and the cost-effectiveness index. By promoting this, the balance between the real economy and medical burden can be continuously evaluated, and policy coordination among stakeholders can be facilitated.

### 2.2. Target Countries and Data

Asia is diverse in terms of health services, geography, and socioeconomic circumstances. In addition, medical systems, actual economic structures, and social and cultural norms vary. Therefore, targeting countries in the Asian region could help solve many of the problems related to UHC, and the findings could be used to derive general solutions applicable to other regions. Thus, this study focused on the Asian region, where both UHC development and economic growth are remarkable. The study sample includes countries with a population of five million or above in 2017, drawn from the Association of Southeast Asian Nations (ASEAN) and neighboring countries with a strong presence in the region. We broadly covered the characteristics (categories) of the target countries by classifying them according to income, as defined by the World Bank.

The study uses the SCI as a proxy of the progress of UHC. The indicators for SCI-related data are “reproductive, maternal, newborn and child health,” “infectious diseases,” “noncommunicable diseases,” and “service capacity and access.” In addition, the country-by-country socioeconomic indicators include “total population,” “population aged 65 and above,” “gross domestic product (GDP) per capita,” “health expenditure per GDP/per capita,” “government health expenditures,” “unemployment rate,” and “poverty rate.” All data were converted into a panel from 2015 to 2017, along with the preparation of SCI-related data and socioeconomic data [20,21,22].

This study estimated the coefficients that define UHC progress and socioeconomics by encompassing and modeling data from multiple countries. However, because UHC progress requires country-specific efforts, as discussed in the introduction, it is also necessary to estimate the coefficients that define UHC progress and socioeconomics for each country. Hence, we conducted a country-specific performance analysis (CEA: country-specific coefficient calculations). Because only a portion of the socioeconomic indicators can be treated in this analysis, we interpreted the calculated coefficients on the premise of the tendency in the panel data analysis.

CEA is often applied to medical-economic evaluations, such as high-priced medicines and health programs, but it can also be applied to macro issues, such as medical systems [19]. In this study, we performed a CEA in a broad sense, using economic level as a cost index and SCI level as an effect index. Income indicators, such as GDP, are not suitable for CEA because the cost index usually deals with expenditures such as medical resource consumption. However, in this study, GDP is closely related to health expenditure, which is a cost index, and it comprehensively embodies economic activities and policy behaviors that affect the progress of UHC; therefore, it was used as a surrogate reference [21,23].

### 2.3. Analysis Method and Conditions

In this study, the interrelationships between SCI and major socioeconomic indicators were analyzed using a multifaceted method. First, we established the relationship between SCI and socioeconomic indicators using Spearman’s rank correlation, multiple regression analysis, and panel data analysis. The objective variable was the SCI, whereas the explanatory variable was the socioeconomic index in the panel data analysis. The fixed effects model, which eliminates individual-specific effects was used. We also applied a logarithmic transformation to adjust the elasticity and distribution characteristics in the regression analysis. To discuss the sensitivity of economic investment to UHC, we performed an ROC curve analysis of health expenditure and the SCI, with a score of 70 points as the criterion.

Considering the theme of this study and the selected data, it was inferred that there was some correlation between the explanatory variables and the error term. Fixed effects models are widely used in social sciences for causal inference using panel data. With that in mind, we selected the basic linear model used in many previous studies as an explanatory model for the panel data analysis [24]. In the analysis, we assumed that the data were balanced and that there were no missing values. We also conducted an ordinary least squares regression (pooled OLS) for reference to verify the analysis results.

_*K*_  

Y _*i,t*_ = α + Σ β _*j*_ X _*j,i,t*_ + μ _*i,t,*_ (1)

^*j =* 1^  

μ *_i,t_* = γ *_i_* + ε *_i,t_* (2)

where Y is the UHC-related index (objective variable) and X is the socioeconomic index (explanatory variable); and **α** is the constant term.

where **γ** is the individual effect and **ε** is the time effect. *i* = 1,* … N* (target country); *t* = 1,* … T* (target period); *j* = 1, *…* *K*; and *K* is the number of explanatory variables.

Subsequently, to contribute to the discussion on SCI of each country, we organized the balance between the degree of achievements related to UHC and the economic level by country. Specifically, a performance analysis based on the concept of the incremental cost-effectiveness ratio (ICER) was conducted using the displacement of SCI achievements and economic levels from 2015 to 2017. In this, performance = difference in outcome (SCI) ÷ difference in the economy (GDP or health expenditure). ICER is a method for discussing the ratio (relative difference) between additional usefulness and increasing cost, used mainly when the dimensions and levels of effect and cost vary between comparisons [25,26]. If the outcome increases and the cost decreases, it becomes a “dominant quadrant” with excellent performance.

   ΔO *_i,t_*    (O *_i,T_* – O _*i*,1_)

P*_i,t_* = ――  =  ――――― (3)

   ΔC *_i,t_*    (C *_i,T_* – C _*i*,1_)

where P is performance as ICER against the baseline, O is social achievement (e.g., UHC progress), and >C is resource consumption (e.g., medical expenses). *i* = 1,* … N* (target country); *t* = 1,* … T* (target period).

The WHO recommends building a framework for UHC according to the actual situation of each country. In this study, we utilized the ratio of displacement of each index over time (incremental cost-effectiveness ratio) to not be affected by the system’s maturity or the scale of the economy. This approach is expected to result in performance evaluations that reflect each country’s specific situation, enabling feedback in the UHC study framework. The SPSS (Ver.26, IBM) was used, and the level of statistical significance was set at 5%. For the panel data analysis, we used the F test.

## 3. Results

### 3.1. Basic Statistical Analysis and Panel Data Analysis

Eleven countries were included in this study. We used the World Bank country income group classification and selected six lower-middle-income countries (LMIC) (54.5%), three upper-middle-income countries (UMIC), and two high-income countries (HIC) (Table 1). 

**Figure 1:**
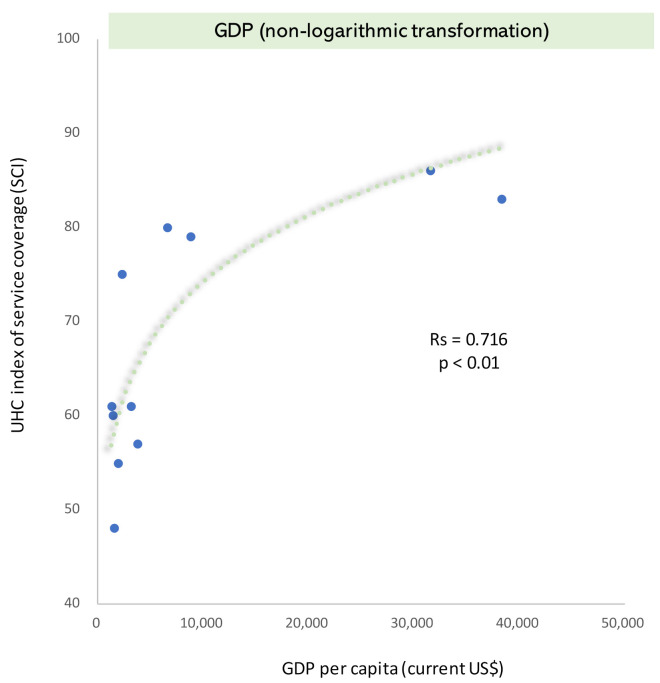

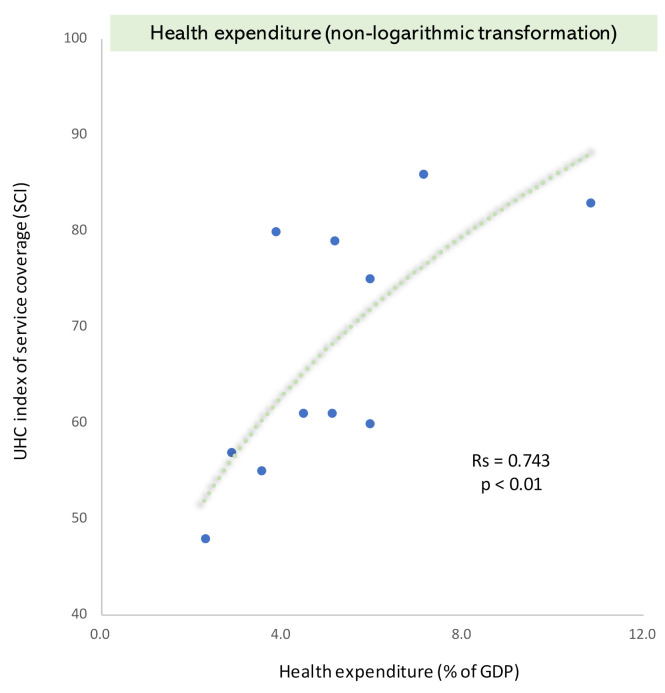
Interrelationship between economic levels (GDP and health expenditure) and SCI levels. Note: UHC, universal health coverage; SCI, service coverage index.

GDP and SCI had a significant positive correlation (Rs = 0.716, *p* < 0.01). Additionally, health expenditure and SCI had a significant positive correlation (Rs = 0.743, *p* < 0.01) (Figure 1). When both GDP and SCI indicators were transformed using logarithms, the tendency did not change significantly (Rs = 0.731, *p* < 0.01) (Figure S1). The results of the panel data analysis showed that the GDP per capita significantly contributed to the SCI (standardized partial regression coefficient, 1.6129; partial regression coefficient, 0.0049; 95% CI, 0.0025–0.0074) (Table 2). The total population, government health expenditure, unemployment rate, and poverty rate were statistically significant, whereas health expenditure was not. The results for the unemployment rate and poverty rate showed a negative trend. The entire model was statistically significant (R^2^ = 0.991, F test: *p* < 0.001). The results of the linear multiple regression analysis using ordinary least squares (pooled OLS) tended to be roughly the same as the results of the panel data analysis (R^2^ = 0.965, F test: *p* < 0.001) (Table S1). However, the health expenditure was significant (standardized partial regression coefficient, 0.6327; partial regression coefficient, 3.6096; 95% CI, 2.2528–4.9664). In the pooled OLS analysis, the variance inflation factor (VIF) was less than 6 for all parameters (GDP = 5.8).

### 3.2. Multiple Regression Analysis of SCI Components

**Figure 2:**
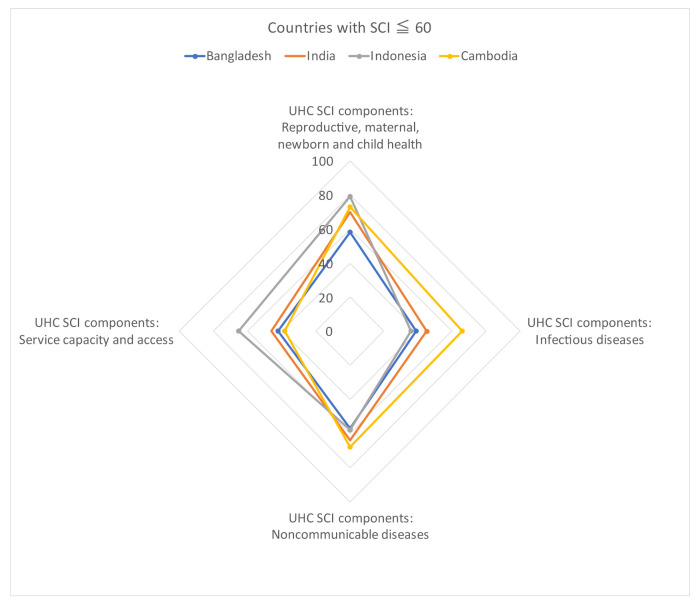

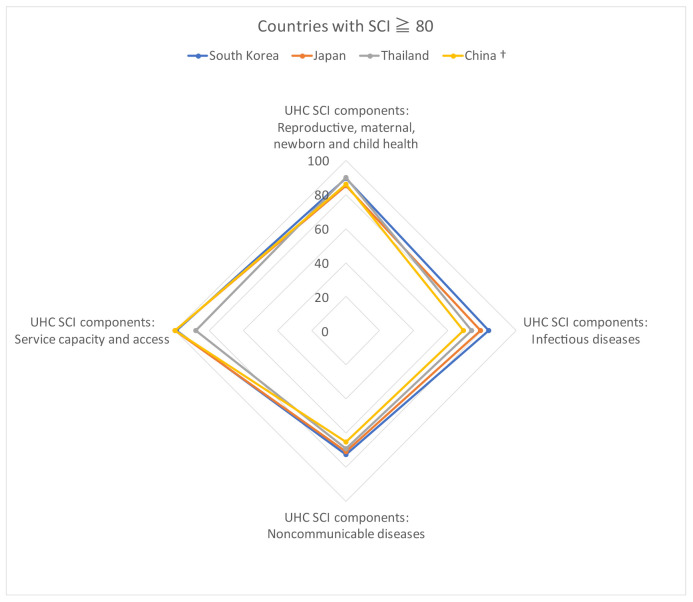
Distribution composition of SCI components by SCI level (60 or below and 80 or above). (†) China has an SCI of 79, but it is shown because it is as close as possible to the relevant group. Note: SCI, service coverage index.

**Figure 3:**
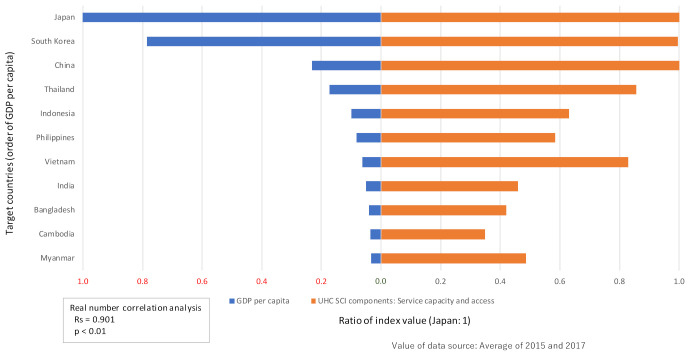
Trends of country-specific economic levels (GDP) and SCI components (service capacity and access). Note: UHC, universal health coverage; SCI, service coverage index.

Each of the four SCI components had different achievement levels (Figure 2). LMIC are the majority among countries with an SCI level of 60 or below (Bangladesh, India, Indonesia, and Cambodia), where “infectious diseases” and “service capacity and access” were more widely dispersed. This was compared to the group of countries with SCIs of more than 80 (South Korea, Japan, Thailand, and China), which were HIC and UMIC. The multiple regression analysis using the annual rate of change in SCI as the objective variable and the annual rate of change in SCI components as the explanatory variable showed that “service capacity and access” was a factor that significantly contributed to the SCI level (standardized partial regression coefficient, 0.9209; partial regression coefficient, 0.3581; 95% CI, 0.3142–0.4019) (Table 3). The ROC curve for health expenditure per GDP for SCI showed a cut-off of 3.7% (*p* < 0.01) for the Youden index and 4.9% (*p* < 0.01) for the shortest distance (AUC = 0.8125, 95% CI: 0.6350–0.9899, *p* < 0.05; Figure S2). Furthermore, when the GDP per capita and “service capacity and access” values of each country were relatively arranged, with Japan as the standard, a positive correlation was observed between the two indicators (single correlation: Rs = 0.901, *p* < 0.01) (Figure 3).

### 3.3. Performance Analysis—Outcome and Economy

**Figure 4:**
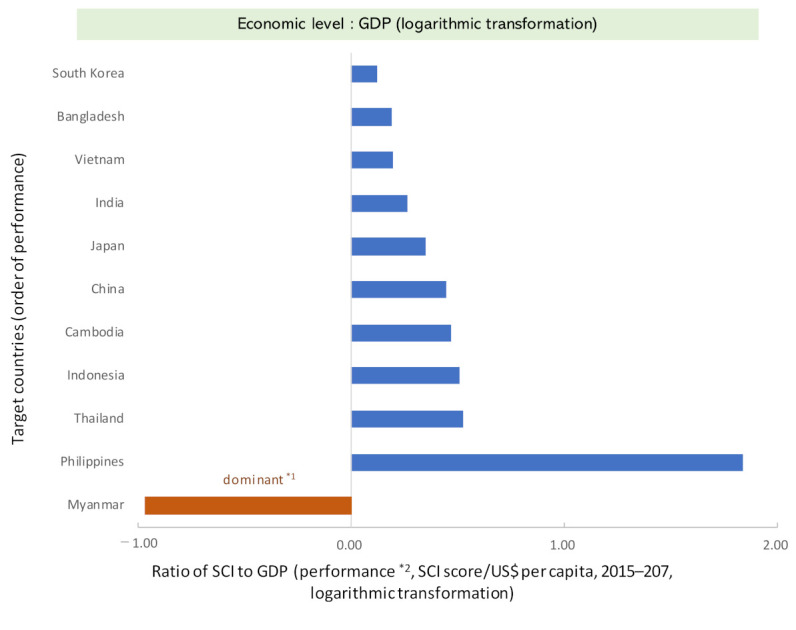

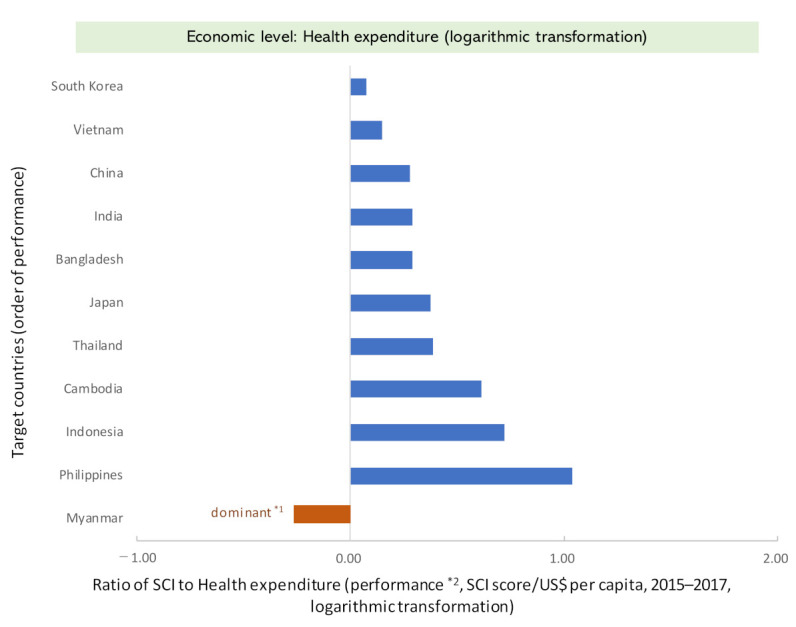
Performance status by country (broad cost-effectiveness analysis, based on displacement from 2015 to 2017). (**Top**) Note: SCI, service coverage index. *1: Dominant is positioned in a more cost-effective dimension with increasing outcomes (SCI) even if the economy (GDP) declines. *2: The performance was a cost-effectiveness analysis (difference in outcome "SCI" ÷ difference in economy "GDP"; displacement from 2015 to 2017). Both indexes were logarithmically transformed in consideration of elasticity. (**Bottom**) Note: SCI, service coverage index. *1: Dominant is positioned in a more cost-effective dimension with increasing outcomes (SCI) even if the economy (Health expenditure) declines. *2: The performance was a cost-effectiveness analysis (difference in outcome "SCI" ÷ difference in economy "Health expenditure"; displacement from 2015 to 2017). Both indexes were logarithmically transformed in consideration of elasticity.

**Figure 5:**
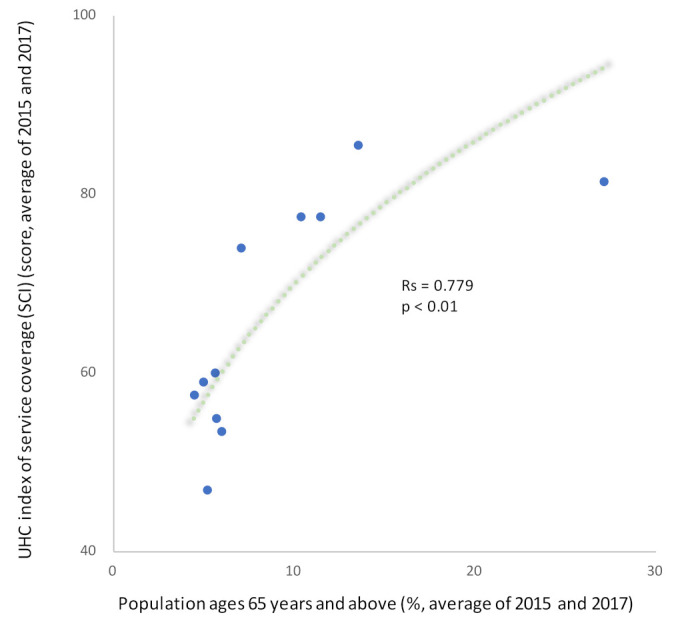

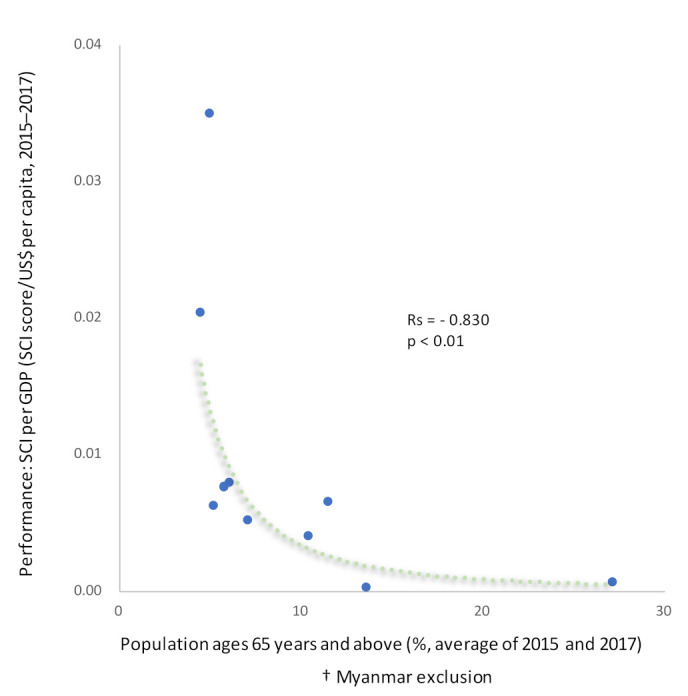
Trends in SCI and performance (economic level: GDP) with respect to the aging rate (percentage of the population aged 65 years and above). (**Top**) Note: UHC, universal health coverage; SCI, service coverage index. (**Bottom**) Note: SCI, service coverage index. (†) Myanmar has a different quadrant (dimension) because it is a "dominant".

Performance analysis after the logarithmic transformation of each index showed that South Korea (HIC) scored the lowest (GDP: 0.12 SCI score/USD per capita, health expenditure: 0.07 SCI score/USD per capita) (Figure 4), while Vietnam (LMIC) and India (LMIC) ranked next. Japan’s (HIC) performance was moderate, and Indonesia (UMIC), Thailand (UMIC), and Cambodia (LMIC) had relatively high performances. The Philippines (LMIC) had the highest performance (GDP: 1.84 SCI score/USD per capita, health expenditure: 1.04 SCI score/USD per capita). In addition, Myanmar (LMIC) was marked as the “dominant quadrant.” In the cost-effectiveness analysis, the quadrant that is more effective but less expensive has the best performance. When the relationship of the proportion of the population aged 65 and above was organized without logarithmic conversion, the SCI score increased with the aging of the population (Rs = 0.779, *p* < 0.01), and performance value decreased (Rs = −0.830, *p* < 0.01) (Figure 5). The relative performance due to health expenditure was similar to that of SCI (Rs = 0.842, *p* < 0.01) (Figure S3).

## 4. Discussion

This study conducted a multifaceted analysis of the SCI to establish the relationship between UHC levels and economic factors. The research approach centered on a panel data analysis and performance analysis (CEA), and the findings obtained from this study do not exist in previous reports. As such, they have novelty and future potential. GDP and government health expenditures were significantly and positively correlated with SCI under a wide range of conditions inherent in the diversity between countries. However, the factors of unemployment and poverty were found to be negatively related to the level of SCI. In addition, “service capacity and access,” which refers to having a medical system with sufficient specialized facilities, medical staff, and health security, significantly contributed to SCI. This factor also had a close relationship with the GDP of each country. A performance analysis based on the ratio of SCI to the GDP/health expenditure showed that South Korea had the lowest performance, while the Philippines had the highest. Although long-term, stable economic growth is desired for UHC progress, the short-term analysis showed that Myanmar fell under the dominant category. Meanwhile, in the case of non-logarithmic conversion, we found that the performance value tended to decrease as aging progressed. Therefore, aging, which is closely related to the maturity of the medical system, also affects UHC [28,29]. 

Health care systems are generally understood to help improve clinical outcomes by increasing public financial investment [30,31]. In fact, the degree of public health expenditures, which can be used as a reference for a country’s health care policy, was positively related to progress toward UHC in this study. In addition, analysis of the ROC curve revealed that the high SIC level (score 70), against the background of a mature medical system can be achieved by increasing the ratio of medical expenses to GDP. However, it has also been reported that the cost effectiveness/performance of the system varies from country to country [32,33]. The results of this study are generally in line with these reports. Therefore, discussions on balance and causality aspects of medical systems and economics are indispensable for further progress toward UHC; that is, the financial mechanism to support UHC and the clinical outcomes that benefit from it are dominated by economic factors. This study also found that the levels of unemployment and poverty, which are distant causes of catastrophic health spending, are factors that lower SCI levels. This suggests that broadly organizing the relationship between UHC and the demographics and maturity of the medical system is desirable, taking into account the trends in economic policy [34,35].

The analysis using a fixed effects model that takes into account the individual effects of each country did not find a significant relationship between UHC progress and the health expenditure indicator (as a total amount). Conversely, in the OLS, which does not take into account impact of these individual effects on explanatory variables, the health expenditure indicator showed a significant relationship, which is in line with previous studies. This difference in results may be due not only to differences in the analysis methods, such as the handling of standard errors in the model, but also to the composition and characteristics of the burden of health care costs. In other words, it is necessary to carefully examine the characteristics of each country’s health care policy that are raised in discussions related to poverty, especially the position of out-of-pocket costs. It is also important to focus on the factors related to the scale of health care financing and its formation process (taxes, insurance premiums) as well as the allocation mechanism. In this study, we found that UHC progress tends to increase as the share of the health care domain in government spending increases. In future studies of UHC development measures, it will also be important to discuss the ideal form of resource allocation (public finance) based on sustainability-based productivity and efficiency or value evaluation (national consensus).

Based on the results of the statistical analysis, there were some cases in which the SCI achievement levels differed, even among countries at the same economic level. Furthermore, in some cases the improvement in SCI was small, even in countries with high economic investment levels. Exploring these factors and considering improvement measures is assumed to promote UHC progress. In this study, the influence of the maturity of the medical system was examined as an additional country-specific factor (instead of the social system, national character, and culture). The results showed that when the level of aging and health expenditure exceeds a certain level, UHC performance will decrease as a country increases the need to raise the UHC goal. In addition, the weight of “service capacity and access” to the SCI was considerable. This secondary index, which embodies the environment of the health care system, can be considered a surrogate index that predicts the maturity of social medical care. The high impact of these factors on UHC means that stable development cannot be expected simply by expanding the scale of spending. This is because of the mechanism related to vital statistics and economic conditions. Thus, policymakers must implement countermeasures based on indicators that can estimate the economic status of the UHC approach, such as cost effectiveness.

The analysis showed that, in some cases, the levels of GDP and SCI were almost the same, but the performance was different: for example, Myanmar (GDP per capita: 1326 USD, SCI: 57.5 score) and Cambodia (GDP per capita: 1200 USD, SCI: 60.0 score) among the LMICs and Japan (GDP per capita: 36,954 USD, SCI: 81.5 score) and South Korea (GDP per capita: 30,169 USD, SCI: 85.5 score) among the HICs (Table 1). However, under limited conditions, the performance of Myanmar was higher than that of Cambodia (although it is a different dimension called “dominant”), while Japan was in the middle range and South Korea in the low range (Figure 4). One reason for these differences was the situation of “service capacity and access.” Cambodia and South Korea tend to be relatively low in this secondary indicator compared to those in the same World Bank country income group (Figure 3). In the case of Myanmar, the influence of the operation of a “health and social care insurance system” and economic trends (flood occurrence) could be related to this difference in performance. In the case of Japan, the existence of a “universal health insurance system” that began in 1958, and other maternal and child health systems may be reasons for this difference.

Long-term research using a more comprehensive range of socioeconomic indicators is desired to promote a more appropriate interpretation and deeper analysis of these phenomena. The development of public medical resources, especially the financial investment system (national burden, insured burden), is indispensable for the sustainable operation of the medical system. Therefore, an analysis of the characteristics of the political system of each country is expected. As rational policy decision-making is imperative for discussing the financial burden, analytical tools such as this study are considered necessary. For example, a CEA could be conducted in future research. In addition, adopting a longitudinal research design would make it possible to account for the effects of fluctuations in external factors, such as the real economy, with high accuracy, although there is a time limit to the preparation of SCI data. However, further consideration is needed on the strengths and weaknesses of the argument using lognormal distribution [36,37].

Declining birth rates, aging population, and maturation of the medical system generally tend to reduce the baseline performance of a medical system as a whole [28,29,38]. However, it is assumed that there is room for countermeasures, including devising population policies and economic measures, in the future; for instance, economic growth strategies could include the promotion of the health care and life sciences industries [39,40]. In addition, improvements in health care programs include disease prevention policies and medical insurance policies [15,41]. This perspective is significant as a point of discussion for the progress of UHC. This study focused on the socioeconomic factors behind these policies and attempted to interpret SCI level fluctuations using multivariate analysis and performance analysis. The results of this research are expected to contribute to future UHC-related discussions, although there are some restrictions.

The main limitations of this study are as follows. First, this study used data from a small number of countries, mainly in Asia, and thus has limited application to discussions around the world. In future research, it is hoped that the model demonstrated here will be verified for extensibility. Next, there was a limit to the comprehensiveness of the socioeconomic indicators, and some discussion remains about the effects of unobserved individual characteristics. In this study, because a fixed effects model was applied, it is necessary to make a more precise comparison with other models and to discuss adjustment content (unobserved time-invariant confounding factors). Furthermore, because the observation period in this study was as short as three years, the reliability of the parameter estimates in the fixed effects model was lowered. There is also concern that the impact of internal and external factors (epidemiology, political affairs, etc.) on the performance analysis (ICER) would be relatively large. In the future, long-term research is expected, in line with the SCI data development.

## 5. Conclusions

The examination of the effects of socioeconomic factors on the development of UHC, GDP, and government health expenditures showed a statistically significant positive correlation of these factors with SCI. However, the unemployment rate and poverty rate had negative relationships with progress toward UHC. In addition, according to the performance analysis, which is broadly a cost-effectiveness analysis, the performance of the Philippines was relatively better than that of other countries, despite its short-term constraints. This approach also suggested that regardless of the maturity of the system or the size of the economy, the status of UHC activities in each country could be evaluated based on the displacement of economic and SCI levels achieved. Long-term research using a broader range of socioeconomic indicators is desired for a more accurate interpretation and deeper analysis of the obtained findings.

## Figures and Tables

**Table 1:** Main overview of eleven countries in the Asian region.

Country	World Bank Country Income Group	Population (Total)	Population Aged 65 and Above		GDP Per Capita	Health Expenditure †
Population > 50 Million People		(Million People)	(% of Total Population)		(Current USD)	(% of GDP)
Bangladesh	LMIC	160	5.1		1406	2.4
Cambodia	LMIC	15	4.4		1326	6.1
China	UMIC	1399	10.3		8397	5.0
India	LMIC	1344	6.0		1793	3.6
Indonesia	UMIC	263	5.7		3627	2.9
Japan	HIC	126	27.1		36,954	10.8
South Korea	HIC	51	13.5		30,169	6.9
Myanmar	LMIC	52	5.6		1200	5.3
Philippines	LMIC	105	4.9		3096	4.4
Thailand	UMIC	69	11.4		6217	3.8
Vietnam	LMIC	95	7.0		2770	5.8
Country	Government health expenditures	Unemployment rate		Poverty rate ††		UHC index of service coverage (SCI)
Population > 50 million people	(% of general government expenditures)	(%: ratio of unemployed persons to the labor force)		(%: poverty gap, the default poverty line is $1.90 PPP/day)		Consists of four indicators
Bangladesh	3.2	4.4		2.3		47.0
Cambodia	6.7	0.4		6.0		57.5
China	9.2	4.0		0.1		77.5
India	3.4	5.5		2.2		53.5
Indonesia	7.4	5.7		0.8		55.0
Japan	23.8	3.2		0.2		81.5
South Korea	4.3	1.2		0.7		85.5
Myanmar	8.0	6.3		1.2		60.0
Philippines	13.1	3.6		0.1		59.0
Thailand	14.6	1.0		0.0		77.5
Vietnam	9.0	2.2		0.4		74.0

GDP, gross domestic product; UHC, universal health coverage; SCI, service coverage index; LMIC, lower-middle-income countries; UMIC, upper-middle-income countries; HIC, high-income countries; Value: average of 2015 and 2017. † Health expenditure was analyzed not only for GDP but also for population (per capita). †† The poverty rate of Cambodia was estimated from 2007 data and poverty ranking [27].

**Table 2:** Panel data analysis of the impact of economic level (GDP and health expenditure, unemployment, poverty) on SCI.

UHC Index of Service Coverage (SCI)	Partial Regression Coefficient	Standardized Partial Regression Coefficient	S.E.	*p*-Value	95% CI
Population (total: million people)	0.0049	0.1921	0.0012	0.0001	0.0025–0.0074
GDP per capita (current USD)	0.0017	1.6129	0.0002	<0.001	0.0013–0.0021
Health expenditure (% of GDP)	2.3481	0.4116	1.5748	0.136	−0.7386–5.4347
Government health expenditures (% of general government expenditures)	1.4511	0.6575	0.2804	<0.001	0.9015–2.0006
Unemployment rate (%: ratio of unemployed persons)	−1.4764	−0.2253	0.7105	0.0377	−2.8689–−0.0838
Poverty rate (%: poverty gap)	−1.6736	−0.2303	0.4674	0.0003	−2.5897–−0.7575
Model: R^2^ = 0.991, F test: *p* < 0.001					

GDP, gross domestic product; UHC, universal health coverage; SCI, service coverage index; S.E., standard error; CI, confidence interval.

**Table 3:** Multiple regression analysis of the contribution of each SCI component to SCI displacement (based on the annual rate of change).

UHC Index of Service Coverage (SCI)	Partial Regression Coefficient	Standardized Partial Regression Coefficient	VIF	*p*-Value	95% CI
UHC SCI components: Reproductive, maternal, newborn and child health	0.5336	0.4370	1.4083	0.0001	0.3826–0.6846
UHC SCI components: Infectious diseases	0.1908	0.6303	3.2725	0.0002	0.1337–0.2478
UHC SCI components: Noncommunicable diseases	0.3701	0.1677	3.4415	0.0780	−0.0566–0.7968
UHC SCI components: Service capacity and access	0.3581	0.9209	1.1719	0.0000	0.3142–0.4019
Constant term	0.0028			0.5974	−0.0095–0.0151
Model: *p* < 0.01					

UHC, universal health coverage; SCI, service coverage index; CI, confidence interval; VIF, variance inflation factor.

## Data Availability

Publicly available datasets were analyzed in this study. The data can be found here: reference numbers [15,16]. Data supporting the findings of the study are available from the corresponding author upon reasonable request.

## Supplementary Materials

The following supporting information can be downloaded at: https://www.mdpi.com/article/10.3390/ijerph19042376/s1. Figure S1: Interrelationship between economic level (GDP) and SCI level (logarithmic transformation, 2017); Figure S2: ROC curve of health expenditure (per GDP: %) for SCI (criterion: score of 70); Figure S3: Interrelationship between GDP-based and health expenditure-based performance. Table S1: Results of linear multiple regression analysis by ordinary least squares (pooled OLS).
