# Supplementary material for: Socioeconomic Determinants of Universal Health Coverage in the Asian Region
Source: Int J Environ Res Public Health. 2022 Feb 18;19(4):2376. doi: 10.3390/ijerph19042376 (PMC8872323; doi:10.3390/ijerph19042376)
Supplement: Supplementary file 1 [file ijerph-19-02376-s001.zip › ijerph-1553794-supplementary.pdf]

Supplementary Files

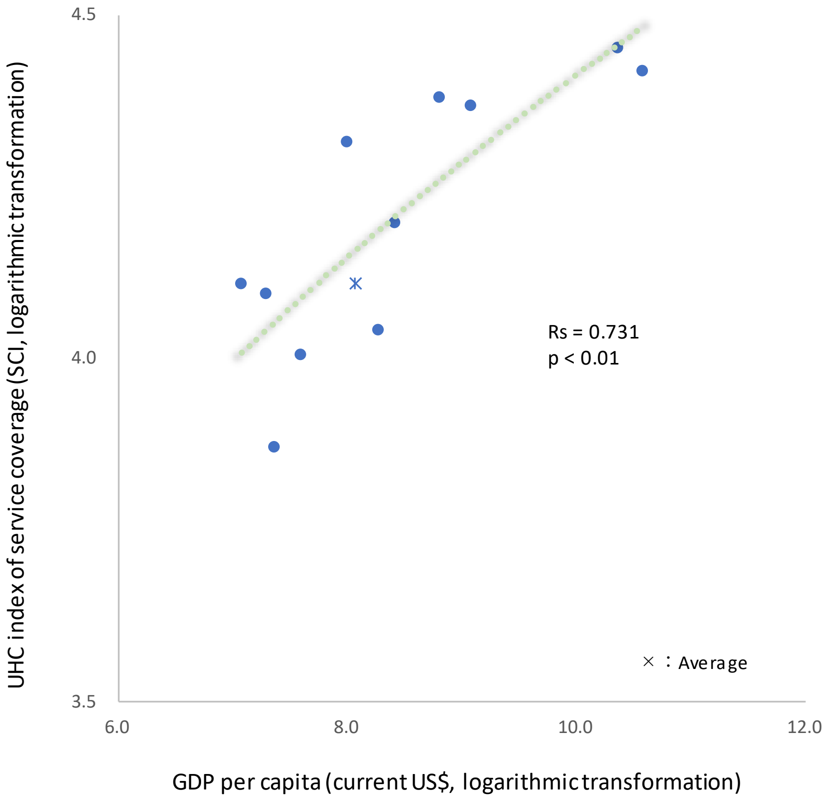

Figure S1. The relationship between economic level (GDP) and SCI level (logarithmic transformation, 2017).

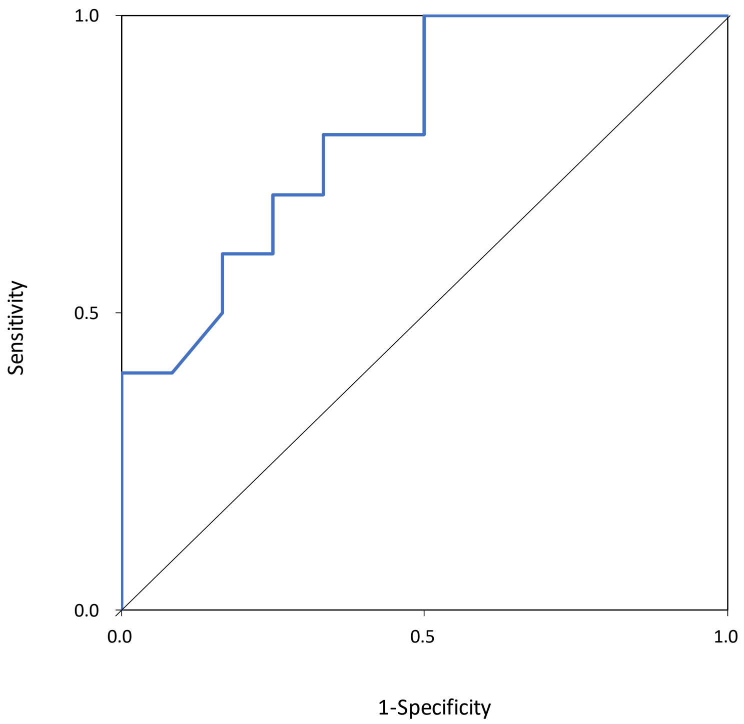

Figure S2. ROC curve of health expenditure (per GDP: %) for SCI (criterion: score of 70).

Supplementary Files

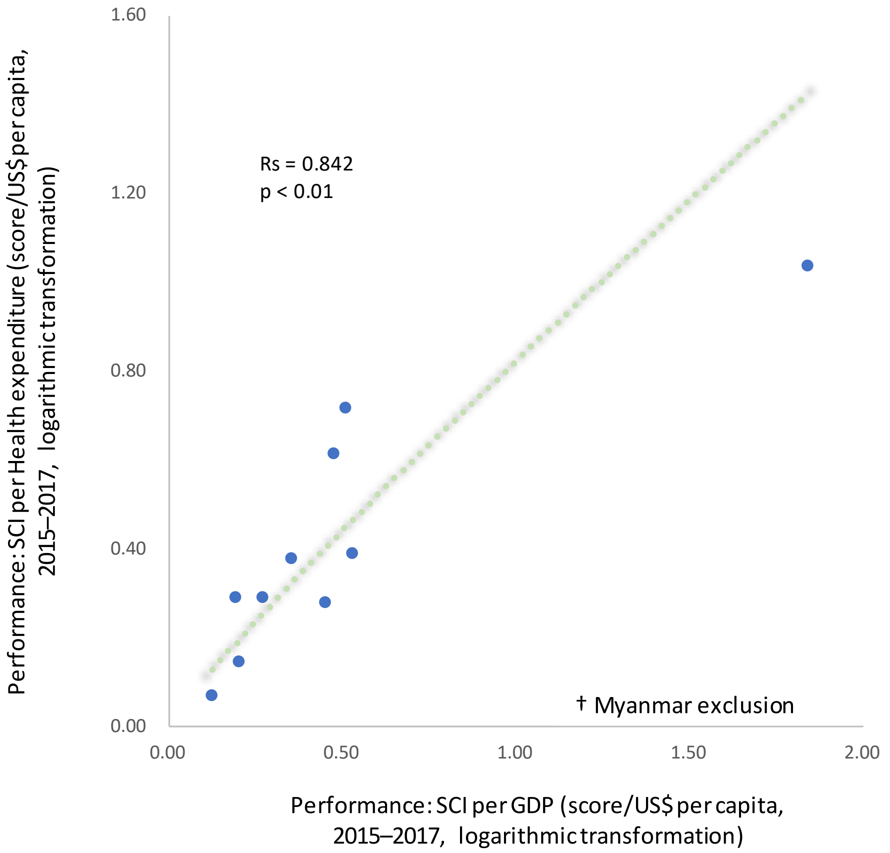

Figure S3. The interrelationship between GDP-based and health expenditure-based performance.

Table S1. Results of linear multiple regression analysis by ordinary least squares (pooled OLS).

UHC index of service coverage (SCI)	Partial regression coefficient	The standardized partial regression coefficient	S.E.	*p*-Value	95% CI
Population (total: million people)	0.0045	0.176	0.0012	0.0018	0.0020 - 0.0071
GDP per capita (current USD)	0.0019	1.7829	0.0002	< 0.001	0.0053 - 0.0023
Health expenditure (% of GDP)	3.6096	0.6327	0.6326	0.0001	2.2528 - 4.9664
Government health expenditures (% of general government expenditures)	1.3082	0.5928	0.2067	< 0.001	0.8648 - 1.7516
Unemployment rate (%: ratio of unemployed persons)	-1.182	-0.1804	0.3832	0.0081	-2.0083 - -0.3602
Poverty rate (%: poverty gap)	-2.134	-0.2937	0.3735	0.0001	-2.9350 - -0.3330
Model: $R^2 = 0.965$, F test: $p < 0.001$					

GDP, gross domestic product; UHC, universal health coverage;
SCI, service coverage index; S.E., standard error; CI, confidence interval.
